# Stress Dynamics in Families with Children with Neuropsychiatric Disorders during the COVID-19 Pandemic: A Three-Year Longitudinal Assessment

**DOI:** 10.3390/jcm12227170

**Published:** 2023-11-18

**Authors:** Ada Claudia Silvana Gruescu, Calin Popoiu, Mihaela Codrina Levai, Sonia Roxana Burtic, Isabella Ionela Sanda, Oana Neda-Stepan, Ovidiu Rosca, Roxana Manuela Fericean, Catalin Dumitru, Lavinia Stelea

**Affiliations:** 1 Department of Pediatrics, Victor Babes University of Medicine and Pharmacy Timisoara, Eftimie Murgu Square 2, 300041 Timisoara, Romania; ada_gruescu@yahoo.com (A.C.S.G.); mcpopoiu@umft.ro (C.P.); 2 Doctoral School, Victor Babes University of Medicine and Pharmacy Timisoara, Eftimie Murgu Square 2, 300041 Timisoara, Romania; dr.soniaburtic@umft.ro (S.R.B.); isabella.sanda@umft.ro (I.I.S.); oanastepan@yahoo.com (O.N.-S.); manuela.fericean@umft.ro (R.M.F.); 3 Research Center for Medical Communication, Victor Babes University of Medicine and Pharmacy Timisoara, Eftimie Murgu Square 2, 300041 Timisoara, Romania; codrinalevai@umft.ro; 4 Department VIII—Neurosciences, Discipline of Psychiatry, “Victor Babes” University of Medicine and Pharmacy, Eftimie Murgu Square 2, 300041 Timisoara, Romania; 5 Department of Infectious Diseases, Victor Babes University of Medicine and Pharmacy, Eftimie Murgu Square 2, 300041 Timisoara, Romania; 6 Department of Obstetrics and Gynecology, Victor Babes University of Medicine and Pharmacy, Eftimie Murgu Square 2, 300041 Timisoara, Romania; dumitru.catalin@umft.ro (C.D.); stelea.lavinia@umft.ro (L.S.)

**Keywords:** COVID-19, child neurology, pandemic, parental stress

## Abstract

Background and Objectives: This study explores the impact of the COVID-19 pandemic on families with children diagnosed with neuropsychiatric disorders, focusing on stress dynamics and quality of life. Materials and Methods: A longitudinal survey was conducted over three years (2020–2022) involving 168 families. The survey included data on demographics, diagnosed conditions, access to therapies, mental well-being, and perceived challenges. Results: The study involved 62, 51, and 55 families in 2020, 2021, and 2022, respectively. ADHD emerged as the most prevalent condition, diagnosed in approximately 32% of the children. The pandemic significantly affected therapy access, with parents reporting a decrease from an average score of 8.1 in 2020 to 6.5 in 2022 (*p* = 0.029). Parents also reported increased feelings of being overwhelmed, peaking at 8.0 in 2021 before declining to 6.3 in 2022 (*p* = 0.017). Despite these challenges, there was a positive trend in family mental well-being, with scores increasing from 5.1 in 2020 to 6.7 in 2022 (*p* = 0.031). The Parental Stress Index (PSI) indicated decreasing trends in Emotional Stress and Parent–Child Communication Difficulties (*p* < 0.001), and Behavioral Challenges in children showed a significant reduction across the years (*p* < 0.001). The Hospital Anxiety and Depression Scale (HADS) reflected a moderate reduction in anxiety levels from 7.6 in 2020 to 6.0 in 2022 (*p* = 0.038), although depression scores did not show a significant change. Conclusions: The COVID-19 pandemic introduced notable challenges for families with neuropsychiatrically diagnosed children, particularly in therapy access and increased parental stress. However, the study also reveals a general improvement in family dynamics, mental well-being, and a decrease in behavioral challenges over time. The necessity of this study stems from the critical need to examine the impact of the COVID-19 pandemic on families with neuropsychiatrically diagnosed children, focusing on their resilience and adaptation in navigating therapy access, parental stress, and overall mental well-being.

## 1. Introduction

Neuropsychiatric disorders in children encompass a wide range of conditions, each with its unique presentation, etiology, and management requirements. Among the most prevalent is Attention Deficit Hyperactivity Disorder (ADHD), affecting a significant proportion of the pediatric population, characterized by inattention, hyperactivity, and impulsivity [1,2]. Autism Spectrum Disorder (ASD) presents challenges in social interactions, communication, and is marked by repetitive behaviors [3]. Pediatric epilepsy with its recurrent seizures, Intellectual Disabilities (ID), mood disorders, anxiety disorders, tic disorders, and other neurodevelopmental conditions contribute to the diverse neuropsychiatric challenges faced by the pediatric population [4,5].

The coronavirus disease 2019 (COVID-19) pandemic, instigated by the severe acute respiratory syndrome coronavirus 2 (SARS-CoV-2), has unequivocally shaken the very foundation of global health, affecting nearly every demographic in its wake [6,7,8,9]. While much attention has been directed towards the physical health implications of the COVID-19, the ramifications on mental and psychosocial health of the pandemic have been profound, especially among families with children diagnosed with neuropsychiatric disorders, among others [10,11]. While the general population grappled with the uncertainty and anxiety triggered by the pandemic, families with children with such disorders faced unique challenges given their children’s heightened vulnerability to stressors and potential exacerbation of symptoms [12,13].

The emerging literature has consistently indicated that neuropsychiatric symptoms can be aggravated by external stressors, particularly in children [14]. The COVID-19 pandemic, with its associated lockdowns, disruptions in routine, and limited access to healthcare and therapeutic interventions, has possibly intensified neuropsychiatric disorders among the affected children and, by extension, their families [15,16]. Preliminary surveys during the early stages of the pandemic reported increased anxiety and stress levels in parents of children with neuropsychiatric disorders given the uncertainty about their child’s health, well-being, and future prospects [17].

Moreover, while the pandemic affected the health of children less severely in terms of physical manifestations compared to that of adults [18,19], its indirect effects—including interruptions in regular care, therapy sessions, and social interactions vital for their psychological well-being—cannot be understated. For families, the dual challenge of navigating the pandemic’s general stressors while ensuring optimal care for their children with neuropsychiatric conditions may have led to elevated stress dynamics, potentially affecting family cohesion, mental well-being, and overall quality of life [20]. In Romania and culturally similar regions, these challenges were compounded by specific pandemic measures, affecting the mental and emotional well-being of these children and their families. Studies such as the one conducted at the University Hospital of Salerno highlight the increased internalizing and externalizing symptoms in children with neuropsychiatric disorders during the pandemic, as well as heightened parental stress [21]. These findings underscore the need for a focused examination of the impact in Romanian and similar contexts, where cultural and healthcare system nuances may influence the experience of these families during such global health crises.

However, the literature presents varied perspectives regarding the resilience and coping mechanisms of such families, and the anxiety and stress levels that they experience. Some studies suggest that families with a history of managing neuropsychiatric disorders may develop adaptive strategies that buffer against external stressors, including pandemics [21,22]. On the contrary, other research points to a heightened risk of exacerbation of symptoms and increased familial stress under such conditions [23,24]. These contradictory findings underscore the complexity of the issue and highlight the need for more targeted research. This study aims to bridge this gap by offering a nuanced understanding of how the pandemic has specifically influenced these families, considering both the potential for increased stress and anxiety and the capacity for resilience. By examining the evolving dynamics over the course of the pandemic, the current research aims to assess the challenges and stress adaptations experienced by families with children suffering from neuropsychiatric disorders by providing a set of specific questionnaires.

## 2. Materials and Methods

### 2.1. Study Design and Ethical Considerations

A longitudinal study was designed to assess the stress dynamics in families with children diagnosed with neuropsychiatric disorders during the three years of the COVID-19 pandemic. This study was conducted in compliance with the guidelines of the Declaration of Helsinki and secured approval from the Ethical Commission of the involved institutions. For this research, background, medical, and neuropsychiatric diagnosis data were primarily extracted from hospital databases at the Emergency Clinical Hospital for Children “Louis Turcanu”, a tertiary pediatric hospital from Timisoara, Romania. The entirety of the questionnaires was administered online to ensure both convenience and safety during the pandemic years. The potential study participants were identified in the hospital database or in private clinics, reached by phone to assess their willingness to participate, and filtered by the following inclusion criteria:

(1) Parents or legal guardians of pediatric patients diagnosed with a neuropsychiatric disorder during the COVID-19 pandemic;
(2) Children diagnosed with conditions such as ADHD, ASD, pediatric epilepsy, ID, mood disorders, anxiety disorders, tic disorders, among others;
(3) Participants able to read, write, and understand the language of the administered questionnaire;
(4) Families that were living together and actively managing the child’s condition during the study years of 2020, 2021, and 2022;
(5) Children between the ages of 1.5 and 5 years old.

Exclusion criteria encompassed:

(1) Families with incomplete contact information or those who did not provide consent;
(2) Participants with cognitive or developmental disabilities potentially impairing their comprehension of the study or ability to accurately complete the questionnaires;
(3) Families wherein the child or any member was diagnosed with COVID-19 at the time of the survey, as this might introduce stressors distinct from the neuropsychiatric disorder;
(4) Families undergoing any severe emotional distress or acute mental health crises potentially skewing the stress dynamics under examination.

Employing a stratified sampling method, 70 families were selected for each year of the pandemic: 2020, 2021, and 2022, totaling 210 families. Of these, 183 families agreed to participate. Online research assistants facilitated the questionnaire administration, and out of the 183, 176 were successfully completed. After excluding those with incomplete responses, 168 were incorporated into the final analysis.

Post data collection, families were stratified into comparison groups based on the severity and type of the child’s neuropsychiatric disorder. The primary grouping involved families of children with behavioral and mood disorders, which were labeled as “less severe/chronic” to assess the more subtle daily stress dynamics. The secondary group consisted of families with children diagnosed with more acute or prominent disorders, such as ASD, ADHD, ID, and pediatric epilepsy, that were labeled as “more severe/acute”. This stratification aimed to delineate the potential variations in stress dynamics across the spectrum of neuropsychiatric conditions.

### 2.2. Questionnaires and Variables

The central aim of this study was to comprehend the stress dynamics within families that have children diagnosed with neuropsychiatric disorders during the three pivotal years of the COVID-19 pandemic. Variables taken into consideration encompassed the age and gender of the child, family’s area of residence, parental marital status, income level, education level, employment status, COVID-19 vaccination status, number of siblings in the household, the specific neuropsychiatric disorder diagnosed, and results from standardized questionnaires such as HADS, PSI, and CBCL 1.5–5 years.

The Hospital Anxiety and Depression Scale (HADS) [21] is a self-reporting instrument constructed to gauge levels of anxiety and depression, particularly within hospital or outpatient contexts. The HADS consists of 14 items, bifurcated into two segments: seven items probing anxiety symptoms (HADS-A) and seven directed towards depression symptoms (HADS-D). Every item avails itself of a 4-point scoring system, wherein higher scores are emblematic of elevated anxiety or depression levels. The scale’s Cronbach’s alpha values are frequently situated between 0.70 and 0.90, contingent upon the population under investigation, testifying to its reliability [19].

The Parenting Stress Index (PSI) [22] is a comprehensive tool to assess and identify high-stress areas in parent–child dynamics. It is instrumental in pinpointing the factors causing parental stress, gauging the magnitude of said stress, and offering insight into potential dysfunctional disciplinary patterns or the development of behavioral issues in children. Its internal consistency and reliability are well-regarded within clinical settings.

The Child Behavior Checklist (CBCL) for Ages 1.5–5 [23] offers a profound evaluation of behavioral and emotional problems in young children. This instrument contains 99 problem items that parents rate concerning their child’s behavior over the past two months. CBCL generates scores on scales that align with the diagnostic categories of DSM-oriented scales, encompassing areas such as Emotionally Reactive, Anxious/Depressed, Somatic Complaints, and others. It stands as a valuable tool for researchers and clinicians alike to unearth potential emotional or behavioral challenges faced by children, and its reliability and validity are robustly established in the literature. Scores below 65 are generally considered within the normal range. Scores between 65 and 69 are considered in the borderline clinical range. This means that the scores are somewhat high but might not be indicative of a significant problem on their own. However, they warrant monitoring and possibly further assessment. Lastly, scores of 70 and above are considered in the clinical pathological range. This indicates a significant level of problems that may require intervention. Scores in this range suggest the behaviors are more problematic and are likely outside of what would be considered typical for children of the same age.

The aforementioned standardized questionnaires were administered online, making it feasible and convenient for participants. Data from the online forms were collected into a spreadsheet after the study period ended. By analyzing the cumulative data, this research endeavored to shed light on the potential correlational patterns between neuropsychiatric disorders in children and the overall familial stress dynamics during the pandemic.

### 2.3. Statistical Analysis

Data management and analysis were conducted utilizing statistical software SPSS version 26.0 (SPSS Inc., Chicago, IL, USA). The sample size was calculated based on a convenience sampling method, with a minimum of 120 respondents on a 95% confidence level and a 10% margin of error. Continuous variables were represented as mean ± standard deviation (SD), while categorical variables were expressed in terms of frequencies and percentages. To analyze the changes between two means, Student’s *t*-test was employed, while for more than two means of continuous variables, the ANOVA test was utilized. The Chi-square test was utilized for categorical variables. A *p*-value threshold of less than 0.05 was set for statistical significance. All results were double-checked to ensure accuracy and reliability.

## 3. Results

### 3.1. Patients’ Background Check

During the COVID-19 pandemic, a total of 62 completed surveys were collected in 2020, 51 in 2021, and 55 families successfully filled the questionnaire in 2022. Considering the data from children, the mean age in 2020 was 3.4 years, which slightly increased to 3.6 years in 2021, and subsequently adjusted to 3.5 years in 2022, without significant differences between groups. When evaluating age distribution, there were no marked disparities in the percentage of children across the four age ranges (1.5–2.5 years, 2.6–3.5 years, 3.6–4.5 years, and 4.6–5 years) over the three years, with the highest prevalence of patients in the 1.5–2.5 years group.

In terms of diagnosed conditions in children, ADHD was consistently the most prevalent condition, with around 32% of children being diagnosed with it across all three years. The distribution of other conditions, including ASD combined with ID, pediatric epilepsy, and mood disorders, also did not show considerable shifts throughout the study period.

Regarding the parents’ data, the mean age increased marginally from 35.6 years in 2020 to 36.9 years in 2022, yet these differences were not statistically significant (*p* = 0.587). The employment status of the parents, spanning employed, unemployed, and self-employed categories, remained relatively stable over the three years, with a non-significant difference. Similarly, the parents’ educational background, categorized as high school, college, and university, did not exhibit significant changes (*p* = 0.596).

An important aspect to note was the percentage of parents who were vaccinated against COVID-19. While no data were available for 2020, there was a marked increase in the vaccination rate from 68.6% in 2021 to 81.8% in 2022, and this change was statistically significant with a *p*-value of 0.012, as described in Table 1.

### 3.2. Analysis of Parental Concerns during the Pandemic

Regarding parent confidence in understanding their child’s specific challenges associated with their neuropsychiatric disorder during the pandemic, the mean scores were relatively consistent across the years, ranging from 6.1 in 2022 to 6.7 in 2021. However, these differences were not statistically significant, as indicated by a *p*-value of 0.362. On the matter of online support resources for families with neuropsychiatric challenges since the onset of the COVID-19 pandemic, parents felt a gradual increase in their availability from 2020 (mean: 6.8) to 2022 (mean: 7.6), although this trend was not found to be statistically significant (*p* = 0.241).

Interestingly, parents perceived a significant decline in their child’s access to necessary therapies or interventions due to the pandemic, with scores decreasing from 8.1 in 2020 to 6.5 in 2022. This decline was statistically significant (*p* = 0.029). Likewise, feelings of being overwhelmed or anxious about the child’s condition during the pandemic also demonstrated a significant change, with the score rising to 8.0 in 2021 before dropping to 6.3 in 2022 (*p* = 0.017). While the impact of feelings of stress or anxiety on parents’ ability to support or care for their children remained relatively stable over the three years (*p* = 0.399), there was a significant positive trend in the overall mental well-being rating of the family since the beginning of the pandemic, with scores increasing from 5.1 in 2020 to 6.7 in 2022 (*p* = 0.031).

Parents’ feelings of support from healthcare professionals during the pandemic hovered around the mid-point, with no statistically significant variation observed across the three years (*p* = 0.512). Similarly, the perceived influence of the family’s mental well-being on addressing the child’s challenges showed an upward trend, albeit not statistically significant (*p* = 0.210). Lastly, the parent sense of being equipped to manage the challenges posed by the child’s neuropsychiatric disorder exhibited a slight downward trend over the years (*p* = 0.481). In contrast, the perceived role of the pandemic in shaping parents’ current perspectives and strategies saw a notable increase, especially in 2022 (mean: 7.9) compared to 2020 (mean: 6.2), a change that was statistically significant (*p* = 0.043), as seen in Table 2.

### 3.3. Analysis of Parental Stress Levels

Table 3 evaluates the PSI survey results stratified by COVID-19 pandemic years. In the facet of Emotional Stress, there was a discernible reduction over the years, with the mean score standing at 61.32 (±22.04) in 2020. This score decreased to 58.29 (±21.38) in 2021 and further dropped to 52.45 (±20.67) in 2022. This continuous decline in scores was statistically significant with a *p*-value of <0.001, suggesting parents experienced a decreasing trend in emotional stress across the years observed. Similarly, the domain of Parent–Child Communication Difficulties also showcased a downward trajectory over the three-year span. The initial score in 2020 was 64.77 (±19.65), which marginally reduced to 62.23 (±19.01) in 2021 and then considerably dropped to 54.33 (±18.59) in 2022. This decline was found to be statistically significant, as indicated by the *p*-value of <0.001, highlighting a perceived improvement in parent–child communication over time.

**Figure 1:**
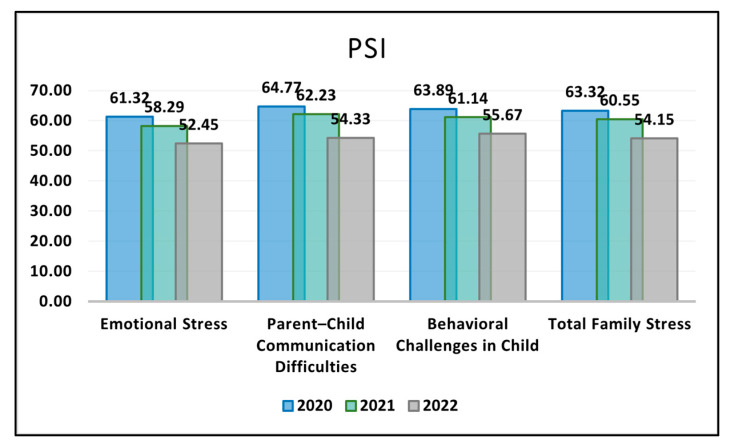
Analysis of the PSI questionnaire results during the COVID-19 pandemic.

In terms of Behavioral Challenges in the Child, there was a decline from an initial score of 63.89 (±21.92) in 2020, moving to 61.14 (±21.28) in 2021, and reaching 55.67 (±21.07) in 2022. The observed decrease over the three years was statistically significant, with a *p*-value of <0.001, reflecting that parents perceived a reduction in their children’s behavioral challenges during the duration of the pandemic. Lastly, the Total Family Stress, which encapsulates the combined influence of all the aforementioned domains, similarly demonstrated a progressive decrease. The score started at 63.32 (±21.20) in 2020, lowered to 60.55 (±20.56) in 2021, and further reduced to 54.15 (±20.11) in 2022, as seen in Figure 1. The overall decrease over the three years was statistically significant with a *p*-value of <0.001, indicating a positive shift in the family stress dynamics among the respondents throughout the period of the COVID-19 pandemic.

### 3.4. Behavioral Analysis of the Children

In 2020, the Emotional Response domain demonstrated a mean score of 70.54 (±12.71), which decreased to 64.40 (±12.63) in 2021 and further declined to 58.45 (±11.10) in 2022. This downward trend was statistically significant with a *p*-value of <0.001, suggesting a reduction in emotional response problems over the years. The domain of Anxiety/Depression also showed a gradual reduction from a mean score of 68.32 (±13.56) in 2020 to 65.21 (±12.73) in 2021, ending at 62.64 (±10.58) in 2022. The overall decline was statistically significant (*p* = 0.044).

Somatic Complaints exhibited a marked decrease from 71.03 (±12.08) in 2020 to 65.29 (±12.47) in 2021 and 59.03 (±9.07) in 2022, with the reduction being statistically significant (*p* < 0.001). However, the Withdrawal domain showed marginal changes, beginning at 66.62 (±13.94) in 2020 and slightly declining to 65.56 (±13.42) in 2021 and 62.58 (±12.90) in 2022. The changes in this domain were not found to be statistically significant (*p* = 0.237). Notably, Sleep Problems, which started at 69.91 (±14.10) in 2020, witnessed a significant reduction by 2022, registering at 61.87 (±12.05), with an intermediate value of 65.49 (±13.49) in 2021. The overall change was significant (*p* = 0.005).

The majority of the other domains, including Aggressive Behavior, Affective Problems, Anxiety Problems, ADHD, and Oppositional-Defiant Problems, displayed similar trends of decline from 2020 to 2022, with their respective changes being statistically significant (all *p*-values < 0.001). However, some domains such as Attention Problems, Pervasive Problems, Internalizing Problems, and Externalizing Problems did not show statistically significant changes over the years. Lastly, the Total Problems score, which serves as an overarching measure of behavioral and emotional challenges, commenced at 71.63 (±18.55) in 2020, reduced to 67.38 (±17.92) in 2021, and settled at 63.54 (±16.55) in 2022. The overall decline was statistically significant with a *p*-value of 0.046, indicating a general improvement in children’s behavioral and emotional status across the pandemic years studied (Table 4).

### 3.5. Analysis of Parental Anxiety during the Pandemic

For the Anxiety component of the HADS, the data revealed that the score began at 7.6 (±3.2) in 2020, decreased to 6.3 (±3.5) in 2021, and further reduced slightly to 6.0 (±4.0) in 2022. This descending pattern in anxiety levels over the years was found to be statistically significant with a *p*-value of 0.038, signifying a moderate reduction in anxiety among the participants during the observed timeframe. In terms of Depression, the scores began at 6.6 (±3.0) in 2020 and showed a decline over the years, registering at 6.1 (±3.3) in 2021 and 5.4 (±3.4) in 2022. However, this decline was not found to be statistically significant, as evidenced by the *p*-value of 0.132.

**Figure 2:**
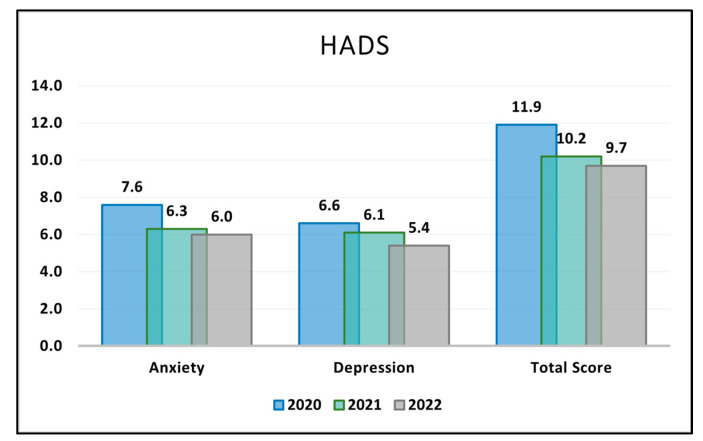
Analysis of the HADS questionnaire results during the COVID-19 pandemic.

The Total Score of the HADS, which combines both anxiety and depression domains, started at 11.9 (±5.5) in 2020. It then decreased to 10.2 (±4.8) in 2021 and slightly further to 9.7 (±5.1) in 2022, as described in Table 5 and Figure 2. The overall decline approached statistical significance with a *p*-value of 0.054, suggesting that there was a general but marginal trend of improvement in the mental well-being of the participants, as measured by the HADS, throughout the duration of the COVID-19 pandemic.

### 3.6. Parental Anxiety and Stress Levels Stratified by Severity of Child Disorder

On evaluating the Parental Stress Index (PSI) scores, it was observed that families with children having more severe or acute disorders generally reported higher levels of stress. For Emotional Stress, the average score was 60.64 (±18.90) for families of children with less severe or chronic conditions, in contrast to 69.38 (±20.03) for the more severe or acute group, a difference which was statistically significant with a *p*-value of 0.039. In terms of Parent–Child Communication Difficulties, the scores were fairly close between the two groups, at 61.77 (±19.36) for the less severe/chronic group and 65.06 (±18.21) for the more severe/acute group, and this difference was not statistically significant (*p* = 0.438). Behavioral Challenges in the child yielded scores of 63.24 (±21.19) for the less severe/chronic group and 68.20 (±17.65) for the more acute group, with the difference not being statistically significant (*p* = 0.279). However, the Total Family Stress scores were significantly different, with the less severe/chronic group scoring 60.32 (±17.92) and the more severe/acute group registering 68.55 (±20.61), evidenced by a *p*-value of 0.042.

For the Hospital Anxiety and Depression Scale (HADS) scores, the trend of higher scores for families with children having more severe or acute disorders continued. In the Anxiety metric, the less severe/chronic group averaged 6.7 (±2.4), whereas the more severe/acute group scored higher at 8.1 (±6.0), a difference that was statistically significant (*p* = 0.045). The scores for Depression were 6.9 (±3.7) for the less severe/chronic families and 7.7 (±5.3) for the more severe/acute group, but this difference did not reach statistical significance (*p* = 0.360). Finally, the combined Total Score of HADS showed that families of children with less severe or chronic disorders scored an average of 10.5 (±6.1), whereas those with more severe or acute conditions reported a score of 12.9 (±7.6). Though the scores for the more severe/acute group were higher, this difference had a *p*-value of 0.087, suggesting it was not statistically significant at the conventional 0.05 level (Table 6).

## 4. Discussion

### 4.1. Literature Analysis

The multifaceted impact of the COVID-19 pandemic on families with children suffering from neuropsychiatric disorders revealed several pivotal insights in this three-year longitudinal assessment. A notable observation from the current study is the perceived decline by parents in their child’s access to vital therapies and interventions, as expressed by previous studies [25,26,27,28]. While various reasons could account for this, lockdowns and restrictions, changes in healthcare prioritization, and disruptions in health services might be prime contributors. The access to critical therapies is a paramount concern as it directly affects the management and progression of neuropsychiatric disorders, underscoring the need for alternative intervention strategies during such global crises [29]. This decline juxtaposes interestingly with the parents’ perception of increased online support resources, highlighting the potential of digital interventions and support during restrictive situations like pandemics. It was previously demonstrated during the past three years of the COVID-19 pandemic that digitalization increased dramatically, including telemedicine services [30].

Emotional dynamics, as reflected by our surveys, seem to be on a positive trajectory facing the end of the pandemic. There was a statistically significant reduction in feelings of being overwhelmed or anxious about the child’s condition over the years and a notable positive trend in overall family well-being. These findings align with the notable downward trend in emotional stress, parent–child communication difficulties, and behavioral challenges. One plausible explanation for these trends could be the extended time families spent together during lockdowns, providing opportunities for bonding, understanding, and mutual support. Nevertheless, previous studies showed that people’s well-being improved with the foreseeing end of the pandemic, and even more so after vaccination programs commenced [31,32].

Yet, the effects of the pandemic were not universally mitigating. While many domains such as aggressive behavior, anxiety problems, and ADHD showed significant declines, others like attention problems and pervasive problems remained relatively constant in our study, indicating that not all challenges were alleviated during this period. This divergence suggests that while the pandemic might have facilitated certain environmental conditions conducive to managing some behavioral problems, inherent neuropsychiatric challenges might remain unchanged, or even exacerbated, without structured interventions.

The contrast between families of children with severe or acute disorders and those with less severe or chronic conditions provides an additional layer of understanding. It is evident that families with children having more severe disorders felt significantly higher stress levels [33], reinforcing the notion that the intensity and nature of the neuropsychiatric condition play a pivotal role in familial stress dynamics. This underlines the importance of tailored therapeutic and counseling interventions based on the severity of the disorder.

Although our study evaluated only families with children affected by neuropsychiatric disorder in the range of 1.5–5 years old, other studies highlighted a marked increase in the severity of mental health issues among hospitalized youth [34]. This was evidenced by a significant rise in psychiatric consultations associated with suicidal ideation, aggression, and eating disorders. There was a near four-fold increase in patient restraints, pointing to worsened behavioral control [35]. Additionally, the greater use of benzodiazepines and antipsychotics might have arisen due to the challenges of infection control measures. While there was an increase in discharges to inpatient psychiatric units, it was influenced by the pressing need to reduce viral transmission, leading to shorter hospital stays. The early pandemic period witnessed a redirection in resources for COVID-19, which could have limited attention to pediatric mental health needs.

Other studies highlighted the psychological toll of lockdown on children with neurodevelopmental disorders and their families [21]. Analysis of the CBCL results revealed a significant deterioration in both internalizing and externalizing symptoms across all participants during the lockdown. This finding was consistent with prior research [36], suggesting that the confinement and disruption of daily routines exacerbated emotional and behavioral issues in these children, especially given their reduced adaptability to new situations.

Focusing on specific conditions, children with ASD aged six and above showed pronounced increases in externalizing, internalizing, and overall issues during the lockdown, in line with previous literature [36]; however, our study did not identify significant differences between these children regarding externalizing and internalizing scores, which might be explained by the age difference. For younger ASD children, under the age of six, there was a decline in most areas, likely due to the absence of distant learning alternatives. Children with ADHD experienced heightened symptoms, such as anxiety, depression, and conduct disorders, mirroring the insights from review [37]. Meanwhile, children in the Epilepsy and SLD groups registered increased scores across all CBCL areas during the pandemic, corroborating several studies [38] that documented the adverse impact of lockdown on these populations.

Nevertheless, the literature presents a complex tapestry of experiences among families with children with neuropsychiatric disorders during the pandemic. While some studies, like that of Operto et al. [21], emphasize the elevated stress and challenges these families faced, others, like that of Shorey et al. [22], acknowledge the resilience and adaptability they might have developed in response to such crises. The variability in these experiences is significant, with some families finding adaptive strategies to manage the new stressors, as suggested by Summers et al. (2021), while others, as highlighted by Italian study [24], experienced exacerbated stress and difficulties in parent–child interactions. This discrepancy not only highlights the diverse impacts of the pandemic on these populations, but also underlines the importance of individualized approaches in understanding and supporting these families. Our findings resonate with these diverse narratives, showing both the challenges and adaptations of families navigating the complexities of neuropsychiatric disorders during a global crisis.

### 4.2. Strengths and Limitations

Some limitations to the study design and methodology warrant mention. Firstly, the study relied on data extracted from a single hospital center, which might limit the generalizability of the results to broader populations or other regions. Similarly, the specificity to Romania might not entirely capture the nuances of familial stress dynamics in different cultural, economic, or healthcare contexts. The use of online questionnaires, while ensuring safety and convenience during the pandemic, may have introduced biases. There is potential for recall bias, especially when parents or guardians were asked to remember and report on past events or feelings, which could have affected the accuracy of the responses. The potential for observer bias also exists, especially given that families were stratifying post data collection based on the perceived severity of the child’s condition.

The age range limitation of children between 1.5 and 5 years old also narrows the scope of the study. Families with children outside this age range, who might have different care needs and stressors, were not captured in this assessment. Exclusion criteria, while necessary to maintain the study’s focus, might have excluded crucial subsets of the population. For instance, excluding families where a member was diagnosed with COVID-19 might overlook the compounded stress of managing a neuropsychiatric disorder while also dealing with the direct impacts of the virus. Lastly, while the study utilized well-regarded tools like HADS, PSI, and CBCL, their application in an online setting without the direct oversight of a clinician might have affected their precision. Participants with cognitive or developmental disabilities that might impair their comprehension were excluded, but it is still possible that nuances or misunderstandings in the questions could introduce response biases.

## 5. Conclusions

Our study reveals the multifaceted and evolving nature of stress dynamics in families with children diagnosed with neuropsychiatric disorders during the COVID-19 pandemic. A key takeaway is the remarkable adaptability and resilience demonstrated by these families despite facing decreased access to essential therapies and heightened initial stress levels. The study highlights a gradual improvement in overall mental well-being, effective parent–child communication, and a reduction in behavioral challenges, underscoring the critical need for continued and targeted support for these families, especially during global crises. These insights emphasize the importance of flexible and accessible healthcare resources and the resilience of families in adapting to unprecedented challenges, offering valuable lessons for healthcare providers and policymakers in supporting these vulnerable groups during times of crisis.

## Figures and Tables

**Table 1:** Background characteristics.

	2020 (*n* = 62)	2021 (*n* = 51)	2022 (*n* = 55)	*p*-Value *
Children’s Data				
Age, years (mean ± SD) **	3.4 ± 1.2	3.6 ± 1.1	3.5 ± 1.3	0.524
Age range				0.451
1.5–2.5	20 (32.3%)	17 (33.3%)	18 (32.7%)	
2.6–3.5	18 (29.0%)	15 (29.4%)	16 (29.1%)	
3.6–4.5	15 (24.2%)	12 (23.5%)	13 (23.6%)	
4.6–5	9 (14.5%)	7 (13.7%)	8 (14.5%)	
Diagnosed Condition				0.482
ADHD	20 (32.3%)	16 (31.4%)	18 (32.7%)	
ASD and ID	12 (19.4%)	11 (21.6%)	10 (18.2%)	
Pediatric epilepsy	10 (16.1%)	9 (17.6%)	8 (14.5%)	
Mood disorders	8 (12.9%)	7 (13.7%)	9 (16.4%)	
Parents’ Data				
Age, years (mean ± SD) **	35.6 ± 6.1	36.2 ± 6.4	36.9 ± 5.8	0.587
Employment Status				0.521
Employed	40 (64.5%)	33 (64.7%)	35 (63.6%)	
Unemployed	15 (24.2%)	12 (23.5%)	13 (23.6%)	
Self-Employed	7 (11.3%)	6 (11.8%)	7 (12.7%)	
Education				0.596
High school	22 (35.5%)	19 (37.3%)	21 (38.2%)	
College	20 (32.3%)	17 (33.3%)	18 (32.7%)	
University	20 (32.3%)	15 (29.4%)	16 (29.1%)	
COVID-19 Vaccinated	-	35 (68.6%)	45 (81.8%)	0.012

* Chi-square or Fisher’s exact test; ** ANOVA test; SD—Standard Deviation; ADHD—Attention Deficit Hyperactivity Disorder; ASD—Autism Spectrum Disorder; ID—Intellectual Disability.

**Table 2:** Unstandardized survey results.

Questions (Answers Given on a Scale from 1 to 10)	2020 (*n* = 62)	2021 (*n* = 51)	2022 (*n* = 55)	*p*-Value *
How confident are you in understanding the specific challenges your child faces due to their neuropsychiatric disorder during the pandemic?	6.5 ± 2.5	6.7 ± 2.9	6.1 ± 2.8	0.362
Since the onset of the COVID-19 pandemic, have you noticed an increase in the availability of online support resources for families with neuropsychiatric challenges?	6.8 ± 3.2	7.2 ± 3.1	7.6 ± 3.0	0.241
To what extent do you believe the pandemic has affected your child’s access to necessary therapies or interventions?	8.1 ± 2.9	7.4 ± 3.0	6.5 ± 3.8	0.029
How often have you felt overwhelmed or anxious about your child’s condition during the pandemic, excluding other personal or work-related stressors?	7.7 ± 3.1	8.0 ± 3.5	6.3 ± 3.6	0.017
To what extent have feelings of stress or anxiety impacted your ability to support or care for your child’s neuropsychiatric needs during the pandemic?	5.5 ± 4.2	5.2 ± 4.1	6.0 ± 4.0	0.399
How would you rate the overall mental well-being of your family since the beginning of the COVID-19 pandemic?	5.1 ± 4.1	6.2 ± 3.0	6.7 ± 2.8	0.031
How supported do you feel by healthcare professionals in addressing your child’s neuropsychiatric challenges during the pandemic?	5.4 ± 3.9	5.7 ± 4.0	5.0 ± 3.7	0.512
To what degree do you believe your family’s mental well-being has influenced your ability to effectively address your child’s neuropsychiatric challenges during the pandemic?	5.0 ± 3.8	5.5 ± 3.7	6.2 ± 3.9	0.210
Considering your understanding prior to the pandemic, how well-equipped do you feel now to manage the challenges posed by your child’s neuropsychiatric disorder?	5.8 ± 3.7	5.6 ± 3.6	5.1 ± 3.9	0.481
How significant a role do you believe the pandemic has played in shaping your current perspectives and strategies for managing your child’s neuropsychiatric challenges?	6.2 ± 4.0	6.5 ± 3.8	7.9 ± 3.9	0.043

* ANOVA test.

**Table 3:** PSI survey results stratified by COVID-19 pandemic years.

PSI (Mean ± SD)	2020 (*n* = 59)	2021 (*n* = 56)	2022 (*n* = 60)	*p*-Value *
Emotional Stress	61.32 ± 22.04	58.29 ± 21.38	52.45 ± 20.67	<0.001
Parent–Child Communication Difficulties	64.77 ± 19.65	62.23 ± 19.01	54.33 ± 18.59	<0.001
Behavioral Challenges in Child	63.89 ± 21.92	61.14 ± 21.28	55.67 ± 21.07	<0.001
Total Family Stress	63.32 ± 21.20	60.55 ± 20.56	54.15 ± 20.11	<0.001

* ANOVA test; SD—Standard Deviation; PSI—Parental Stress Index (Higher scores on the PSI indicate greater levels of stress).

**Table 4:** CBCL survey results stratified by COVID-19 pandemic years.

CBCL (Mean ± SD)	2020 (*n* = 59)	2021 (*n* = 56)	2022 (*n* = 60)	*p*-Value *
Emotional Response	70.54 ± 12.71	64.40 ± 12.63	58.45 ± 11.10	<0.001
Anxiety/Depression	68.32 ± 13.56	65.21 ± 12.73	62.64 ± 10.58	0.044
Somatic Complaints	71.03 ± 12.08	65.29 ± 12.47	59.03 ± 9.07	<0.001
Withdrawal	66.62 ± 13.94	65.56 ± 13.42	62.58 ± 12.90	0.237
Sleep Problems	69.91 ± 14.10	65.49 ± 13.49	61.87 ± 12.05	0.005
Attention Problems	70.93 ± 15.63	67.00 ± 14.35	65.92 ± 13.60	0.146
Aggressive Behavior	72.22 ± 16.32	68.34 ± 15.30	64.20 ± 14.28	<0.001
Affective Problems	71.63 ± 14.00	66.19 ± 13.50	62.58 ± 12.90	<0.001
Anxiety Problems	72.54 ± 14.32	67.69 ± 13.12	62.51 ± 12.32	<0.001
Pervasive Problems	68.74 ± 15.16	66.29 ± 14.54	64.73 ± 13.11	0.307
ADHD	70.26 ± 14.39	66.94 ± 13.11	63.24 ± 12.35	<0.001
Oppositional-Defiant Problems	70.09 ± 14.24	66.96 ± 13.76	63.05 ± 12.23	<0.001
Internalizing Problems	69.71 ± 16.50	68.44 ± 15.86	64.67 ± 14.50	0.190
Externalizing Problems	67.04 ± 17.11	65.60 ± 16.03	65.01 ± 15.10	0.779
Total Problems	71.63 ± 18.55	67.38 ± 17.92	63.54 ± 16.55	0.046

* ANOVA test; SD—Standard Deviation; CBCL—Child Behavior Checklist (A higher score indicates a greater degree of problems or challenges in that particular behavioral or emotional area); ADHD—Attention Deficit Hyperactivity Disorder.

**Table 5:** HADS survey results stratified by COVID-19 pandemic years.

HADS (Mean ± SD)	2020 (*n* = 59)	2021 (*n* = 56)	2022 (*n* = 60)	*p*-Value *
Anxiety	7.6 ± 3.2	6.3 ± 3.5	6.0 ± 4.0	0.038
Depression	6.6 ± 3.0	6.1 ± 3.3	5.4 ± 3.4	0.132
Total Score	11.9 ± 5.5	10.2 ± 4.8	9.7 ± 5.1	0.054

* ANOVA test; SD—Standard Deviation; HADS—Hospital Anxiety and Depression Scale (higher scores indicate greater levels of anxiety or depression).

**Table 6:** Survey results stratified by children with behavioral and mood disorders, and those with more acute or prominent disorders, such as ASD or pediatric epilepsy.

Scores (Mean ± SD)	Less Severe/Chronic (*n* = 144)	More Severe/Acute (*n* = 24)	*p*-Value *
PSI (mean ± SD)			
Emotional Stress	60.64 ± 18.90	69.38 ± 20.03	0.039
Parent–Child Communication Difficulties	61.77 ± 19.36	65.06 ± 18.21	0.438
Behavioral Challenges in Child	63.24 ± 21.19	68.20 ± 17.65	0.279
Total Family Stress	60.32 ± 17.92	68.55 ± 20.61	0.042
HADS (mean ± SD)			
Anxiety	6.7 ± 2.4	8.1 ± 6.0	0.045
Depression	6.9 ± 3.7	7.7 ± 5.3	0.360
Total Score	10.5 ± 6.1	12.9 ± 7.6	0.087

* Student’s *t*-test; SD—Standard Deviation; less severe/chronic—families of children with behavioral and mood disorders; more severe/acute—families with children diagnosed with more acute or prominent disorders, such as ASD, ADHD, ID, and pediatric epilepsy; HADS—Hospital Anxiety and Depression Scale (higher scores indicate greater levels of anxiety or depression; PSI—Parental Stress Index (higher scores on the PSI indicate greater levels of stress).

## Data Availability

The data presented in this study are available on request from the corresponding author.
